# THE ELECTRONIC FRAILTY INDEX BASED ON THE COMPREHENSIVE GERIATRIC ASSESSMENT: DEVELOPMENT AND TESTING

**DOI:** 10.1093/geroni/igz038.2529

**Published:** 2019-11-08

**Authors:** Betty Chinda, Katayoun Sepehri, Macy Zou, Mckenzie Braley, Antonina Garm, Grace Park, Kenneth Rockwood, Xiaowei Song

**Affiliations:** 1 Department of Biomedical Physiology and Kinesiology, Simon Fraser University, Burnaby, British Columbia, Canada; 2 Health Research and Innovation, Surrey Memorial Hospital, Fraser Health Authority, Surrey, British Columbia, Canada; 3 Community Actions and Resources Empowering Seniors, Fraser Health Authority, British Columbia, Canada, Canada; 4 Community Actions and Resources Empowering Seniors, Fraser Health Authority, Surrey, British Columbia, Canada; 5 Division of Geriatric Medicine, Dalhousie University, Halifax, Nova Scotia, Canada

## Abstract

Frailty is characterized by loss of biological reserves across multiple systems and associated with increased risks of adverse outcomes. A Frailty Index (FI) constructed using items from the Comprehensive Geriatric Assessment (CGA) has been validated in geriatric medicine settings to estimate the level of frailty. Traditionally, the CGA used a paper form and the CGA-based FI calculation was a manual process. Here, we reported building of an electronic version of the assessment on personal computers (PC), i.e., standalone eFI-CGA, to benefit frailty assessment at points of care. The eFI-CGA was implemented as a software tool on the WinForms platform. It automated the FI calculation by counting deficits accumulation across multiple domains assessing medical conditions, cognition, balance, and dependency of activities of daily living. Debugging, testing, and optimization were performed to enhance the software performance with respect to automation accuracy (processing algorithm), friendly user interface (user manual and feedback), and data quality control (missing data and value constraints). Systematically-designed simulation dataset and anonymous real-world cases were both applied. The optimized assessment tool resulted in fast and convenient conductance of the CGA, and a 100% accuracy rate of the eFI-CGA automation for up to four decimals. The stand-alone eFI-CGA implementation has provided a PC-based software tool for use by geriatricians and primary and acute care providers, benefiting early detection and management of frailty at points of care for older adults.

